# A subset of cancer cell lines is acutely sensitive to the Chk1 inhibitor MK-8776 as monotherapy due to CDK2 activation in S phase

**DOI:** 10.18632/oncotarget.6364

**Published:** 2015-11-22

**Authors:** Nandini Sakurikar, Ruth Thompson, Ryan Montano, Alan Eastman

**Affiliations:** ^1^ Department of Pharmacology and Toxicology, and Norris Cotton Cancer Center, Geisel School of Medicine at Dartmouth, Lebanon, NH, USA

**Keywords:** cell cycle checkpoint, DNA damage response, Chk1, Wee1, CDK2

## Abstract

DNA damage activates Checkpoint kinase 1 (Chk1) to halt cell cycle progression thereby preventing further DNA replication and mitosis until the damage has been repaired. Consequently, Chk1 inhibitors have emerged as promising anticancer therapeutics in combination with DNA damaging drugs, but their single agent activity also provides a novel approach that may be particularly effective in a subset of patients. From analysis of a large panel of cell lines, we demonstrate that 15% are very sensitive to the Chk1 inhibitor MK-8776. Upon inhibition of Chk1, sensitive cells rapidly accumulate DNA double-strand breaks in S phase in a CDK2- and cyclin A-dependent manner. In contrast, resistant cells can continue to grow for at least 7 days despite continued inhibition of Chk1. Resistance can be circumvented by inhibiting Wee1 kinase and thereby directly activating CDK2. Hence, sensitivity to Chk1 inhibition is regulated upstream of CDK2 and correlates with accumulation of CDC25A. We conclude that cells poorly tolerate CDK2 activity in S phase and that a major function of Chk1 is to ensure it remains inactive. Indeed, inhibitors of CDK1 and CDK2 arrest cells in G1 or G2, respectively, but do not prevent progression through S phase demonstrating that neither kinase is required for S phase progression. Inappropriate activation of CDK2 in S phase underlies the sensitivity of a subset of cell lines to Chk1 inhibitors, and this may provide a novel therapeutic opportunity for appropriately stratified patients.

## INTRODUCTION

In an undamaged cell, progression through G1, S and G2 phase of the cell cycle is dependent on temporal activation of cyclin-dependent kinases CDK1 and CDK2 in complex with cyclins E, A and B. CDK1/2 usually exist in a phosphorylated and inactive form that requires dephosphoryation for activation at an appropriate time in the cell cycle. The inhibitory phosphorylation on tyrosine 15 and threonine 14 is catalyzed by Wee1 or Myt1 (also known as PKMyt1). The subsequent dephosphorylation and activation of CDK1/2 is mediated by one of three CDC25 phosphatases (A, B or C) [1].

Many anticancer agents damage DNA thereby activating a cell cycle checkpoint that arrests cell cycle progression and permits repair and recovery. The arrest requires activation of Checkpoint kinase 1 (Chk1) that inhibits CDC25 and thereby prevents activation of CDK1/2 [2]. Consequently, Chk1 inhibitors have been developed as potential adjuvants to DNA damaging agents as they circumvent arrest before repair is complete, drive cells through the cell cycle, and increase cell killing [2]. In addition, antimetabolites such as gemcitabine and hydroxyurea deprive cells of deoxyribonucleotides thereby stalling replication. These stalled replication forks are stabilized by Chk1, such that inhibition of Chk1 results in replication fork collapse and DNA double-strand breaks (DSB) [3, 4]. Wee1 inhibitors have also been shown to enhance DNA damage-induced cell killing [5, 6]. Whether either of these approaches can elicit cytotoxicity that is selective for the tumor cells remains to be established, although growth suppression in tumor xenografts suggests these approaches are tolerated [4, 7].

Recently, both Chk1 and Wee1 inhibitors have been shown to have single agent activity in some cell lines, while the combination of these inhibitors has been reported to induce synergistic killing [3, 8-10]. We recently reported that U2OS cells are very sensitive to short incubation with a low concentration of the Chk1 inhibitor MK-8776 [11]. The DSB that occur in S phase are the result of Mus81-mediated cleavage of DNA, which can be prevented by inhibiting the single-strand nuclease activity of Mre11, which in turn is dependent on CDK1/2 [11]. Here, we set out to determine the extent of sensitivity in a large panel of cell lines and to define the specific CDK that is involved in the induction of DSB in cells sensitive to MK-8776. This necessitated a critical reanalysis of the methods that discriminate CDK1 from CDK2.

Tumors frequently exhibit oncogene-induced replicative stress, and it has been suggested that this may provide a therapeutic opportunity to selectively target such cells, in particular through inhibition of Chk1 or its upstream activator kinase ataxia telangiectasia-mutated and Rad3-related (ATR) [12, 13]. This would suggest a large proportion of tumors should be sensitive to Chk1 inhibition, yet this is not the case. Only a few cell lines are sensitive to MK-8776 as a single agent. In contrast, the majority of cell lines are sensitive to the Wee1 inhibitor AZD1775 (formerly known as MK-1775). It has also been suggested that DNA damage induced by either Chk1 or Wee1 inhibition results from aberrant mitotic entry [5, 14], yet this is also inconsistent with the observations reported here. The results suggest that only a subset of cell lines activate CDK2 in S phase upon incubation with a Chk1 inhibitor, and that this might provide a chemical synthetic lethal interaction whereby a subset of tumors will respond to Chk1 inhibitors as monotherapy.

## RESULTS

### Differential sensitivity of cells to MK-8776 and AZD1775

**Figure 1:**
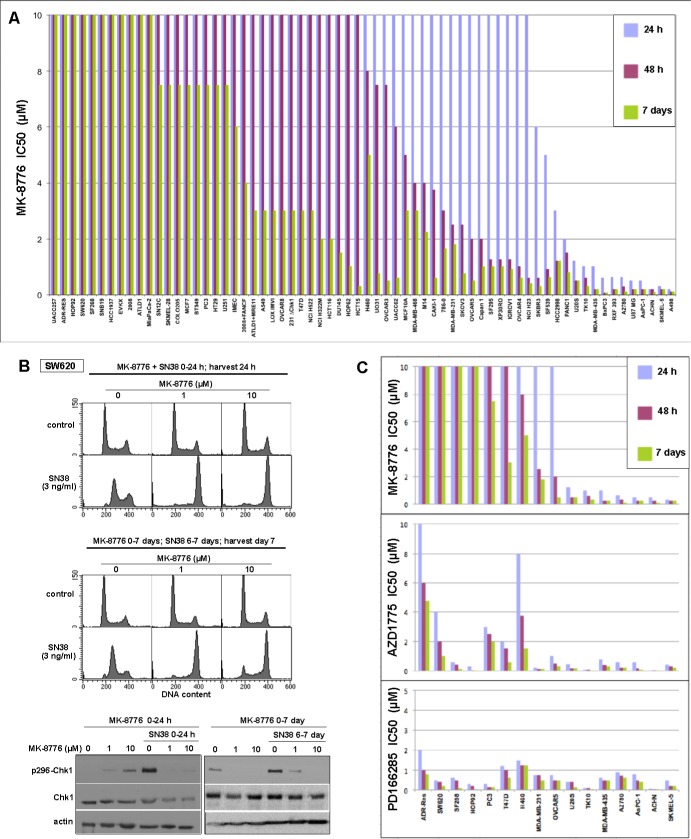
Sensitivity of cell lines to Chk1 and Wee1 inhibitors **A.** Each cell line was incubated with 0-10 μM MK-8776 for either 24 h or 48 h, the drug was removed, and cells incubated in fresh media for an additional 5-6 days. Alternately, cells were incubated in drug continuously for 6-7 days. Cells were lysed in the well, stained with Hoechst 33258, and the concentration that inhibited growth by 50% was recorded. Each histogram reflects a single 96-well cytotoxicity assay, but sensitivity and resistance of selected cell lines was confirmed in subsequent experiments. **B.** SW620 cells were incubated with 3 ng/ml SN38, alone or concurrently with 1 or 10 μM MK-8776 for 24 h. Cells were harvested and analyzed by flow cytometry (top). Parallel cultures were seeded at low density, incubated with 0, 1 or 10 μM MK-8776 for 7 days, and SN38 was added for the final 24 h (middle). Similarly treated cells were also lysed and analyzed for Chk1 autophosphorylation by western blotting (bottom). **C.** A subset of the cell lines was incubated with MK-8776 (data from panel A), AZD1775 or PD166285 and analyzed as described in panel **A.**

We recently demonstrated that several cell lines are acutely sensitive to the Chk1 inhibitor MK-8776 as a single agent [3, 4, 11]. We have expanded this analysis to a large panel of cell lines (Figure 1A). In this cytotoxicity screen, cells were incubated with drug for either 24 or 48 h, then the drug was removed and incubation continued to 7 days. Alternately, cells were incubated continuously for 7 days. Cytotoxicity observed after the shorter incubations reflects the inability of the cells to recover after a potentially toxic insult. We show that 11 cell lines are very sensitive (IC50 < 2 μM) to short incubation with MK-8776, while another 9 cell lines are sensitive to this concentration after 48 h incubation. However, we now show that the majority of cell lines are resistant (IC50 > 10 μM) and many continue to grow even when MK-8776 is left in the media for the entire 7 days (Figure 1A). These values are comparable to the plasma concentrations of MK-8776 in patients where a concentration of > 1 μM was maintained for at least 6 h [15].

Resistance to MK8776 does not appear to be due to lack of drug bioavailability or defects in Chk1 as these cell lines can still be sensitized to either hydroxyurea or gemcitabine when incubated with MK-8776 [3, 4]. To confirm that Chk1 remained inhibited in the resistant cells for a long period, we investigated the impact of MK-8776 on response of cells to the topoisomerase I inhibitor SN38 as previously studied [3, 16]. SW620 cells incubated with SN38 for 24 h arrested in S phase, but when incubated concurrently with SN38 and either 1 or 10 μM MK-8776, the cells arrested in G2, consistent with abrogation of S phase arrest (Figure 1B). Parallel cell cultures were plated at low density in the presence of 0, 1 or 10 μM MK-8776 and allowed to grow for 6 days at which time SN38 was added for the final 24 h. In the absence of MK-8776, SN38 arrested the cells in S phase as expected. However, cells that had been incubated for 6 days in MK-8776 failed to arrest in S phase when SN38 was added, but rather arrested in G2 consistent with concurrent Chk1 inhibition. A second indication of the activity of Chk1 is its autophosphorylation on ser296 that is observed after a 24-h incubation with SN38 (Figure 1B). Concurrent or 6-day pretreatment with MK-8776 prevented this phosphorylation. Similar results were obtained with other resistant cell lines (e.g., ADR-Res and MiaPaCa2; data not shown). These experiments demonstrate that MK-8776 continued to inhibit Chk1 for at least 7 days yet these resistant cells continued to proliferate.

A subset of cell lines was selected for further analysis, and compared for their sensitivity to the Wee1 inhibitor AZD1775 (Figure 1C). While the majority of cell lines appeared to be sensitive to a short incubation with AZD1775, a few cell lines appeared to be more resistant even to continuous exposure. Importantly, most cell lines that were resistant to MK-8776 were sensitive to AZD1775. As discussed further below, the cell lines resistant to AZD1775 were sensitive to incubation with the broad spectrum tyrosine kinase inhibitor PD166285 which inhibits both Wee1 and Myt1 [17] (Figure 1C).

### Differential role of CDK1 and CDK2 in regulating mitosis and DNA double-strand breaks

**Figure 2:**
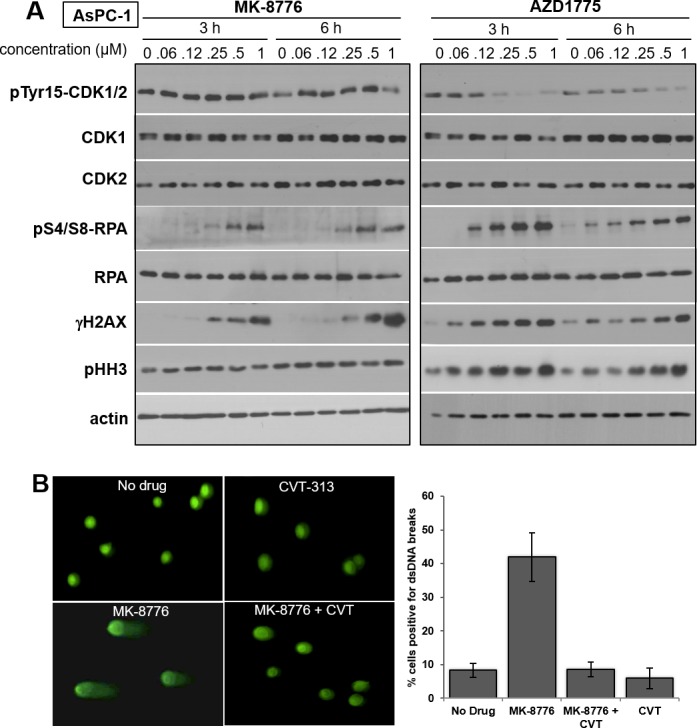
Impact of MK-8776 and AZD1775 on markers of CDK1/2 activity and DNA double-strand breaks **A.** AsPC-1 cells were incubated with the indicated concentrations of MK-8776 and AZD1775 for 3 or 6 h, then lysed and analyzed by western blotting for the indicated proteins. **B.** AsPC-1 cells were incubated with 2 μM MK-8776 for 6 h and analyzed by the comet assay for the appearance of DNA double-strand breaks. Parallel cultures were incubated with MK-8776 plus 5 μM CVT-313. Results are expressed as the percent of cells with an d = increase tail moment greater than 85% of the control cells (i.e., 1 SD). The experiment was performed in triplicate (mean +/− SE).

**Figure 3:**
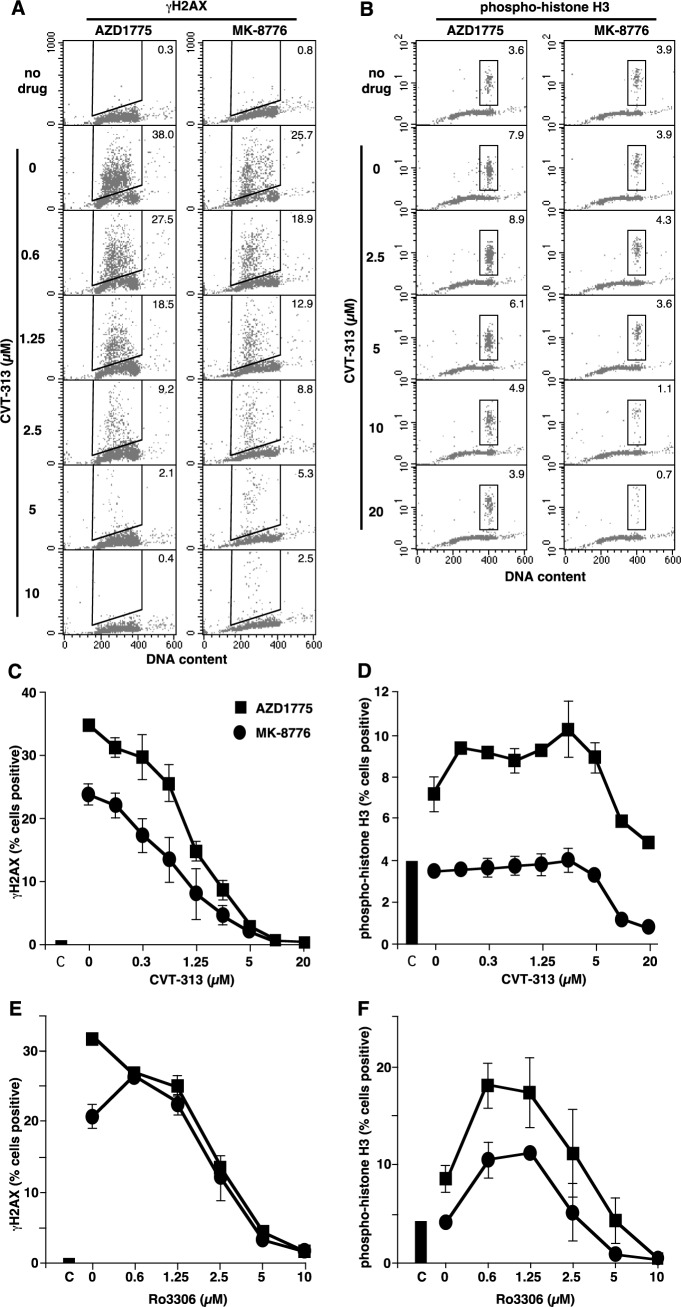
Induction of γH2AX and pHH3 by MK-8776 and AZD1775, and suppression by inhibitors of CDK1 and CDK2 **A.** and **B.** AsPC-1 cells were incubated with 2 μM MK-8776 or AZD1775 for 6 h alone (row 2) or with the further addition of the indicated concentrations of CVT-313, then analyzed by 2-dimensional flow cytometry for DNA content and either γH2AX **A.** or pHH3 **B.** Cells in the top row received no drug. The inset numbers reflect the percent of cells positive for either γH2AX or pHH3. **C.** and **D.** The percent of cells positive for γH2AX **C.** or pHH3 **D.** in triplicate experiments are reported (mean +/− SE). **E.** and **F.** Parallel experiments were performed in which Ro3306 was used to suppress γH2AX **E.** and pHH3 **F.** induced by MK-8776 and AZD1775.

The following experiments were designed to investigate the mechanisms underlying the differential response of cells to MK-8776 and AZD1775. We initially studied the pancreas tumor cell line AsPC-1 which is acutely sensitive to both drugs, and investigated the concentration and time required to induce phosphorylation of RPA and H2AX (this phosphorylation on ser139 is known as γH2AX) which are commonly used as markers of single-stranded DNA and DSB, respectively (Figure 2A). γH2AX is occasionally reported to occur in the absence of DSB although this might be explained by the recent observation that stalled replication forks can rapidly regress giving a “one-sided DSB” [18]. We previously reported that MK-8776 induces γH2AX in U2OS cells that is associated with the appearance of DSB in the comet assay [11]. Here, we demonstrate the appearance of DSB in MK-8776-treated AsPC-1 cells as well (Figure 2B). The proportion of cells positive for DSB correlated with the proportion of cells positive for γH2AX by flow cytometry (Figure 3). We also observed concurrent appearance of S4/S8-phosphorylated RPA, a substrate of DNA-protein kinase, which in turn is activated by DSB (Figure 2A).

Incubation of AsPC-1 cells with ≥125 nM AZD1775 for 3 or 6 h caused dephosphorylation of tyrosine 15-CDK1/2, and the appearance of both γH2AX and phospho-RPA (Figure 2A). There was also an increase in phospho-histone H3 (pHH3), a common marker for mitotic cells. In contrast, MK-8776 induced minimal dephosphorylation of CDK1/2 or increase in pHH3, but it still induced γH2AX and phospho-RPA at 250 - 500 nM. Very similar results were obtained in another sensitive cell line, ACHN (Supplemental Figure S1).

To further contrast the difference between MK-8776 and AZD1775, we performed 2-dimensional flow cytometry to assess the phase of the cell cycle at which γH2AX and pHH3 appeared. γH2AX was induced in S phase cells by both drugs (Figure 3A; compare rows 1 and 2). AZD1775 was about 2-fold more potent than MK-8776, and the majority of S phase cells exhibit γH2AX within 6 h (Supplemental Figure S2). A time course experiment with MK-8776 demonstrated that, as cells continue to enter S, they accumulate in early S phase with high γH2AX (Supplemental Figure S2D). While a small proportion of untreated cells exhibited pHH3, these were all in G2/M consistent with a few cells passing through mitosis. Incubation with AZD1775 but not MK-8776 induced a significant increase in pHH3 that was only observed in the G2/M population (Figure 3B; compare rows 1 and 2), and is consistent with activation of the mitotic CDK1 (and presumably of aurora kinase B which is the ultimate kinase that phosphorylates pHH3; [19]). It is important to emphasize that all the γH2AX-positive cells are in S phase and so are distinct from the pHH3-positive cells. Hence, the appearance of γH2AX is not a consequence of premature mitosis or mitotic catastrophe. These observations highlight a significant difference between the two drugs as MK-8776 does not appear to activate CDK1. We and others have previously reported that inhibition of CDK1/2 can prevent Chk1-inhibitor-induced γH2AX [11, 20], thereby suggesting that CDK2 is probably responsible for the induction of γH2AX in S phase cells.

**Figure 4:**
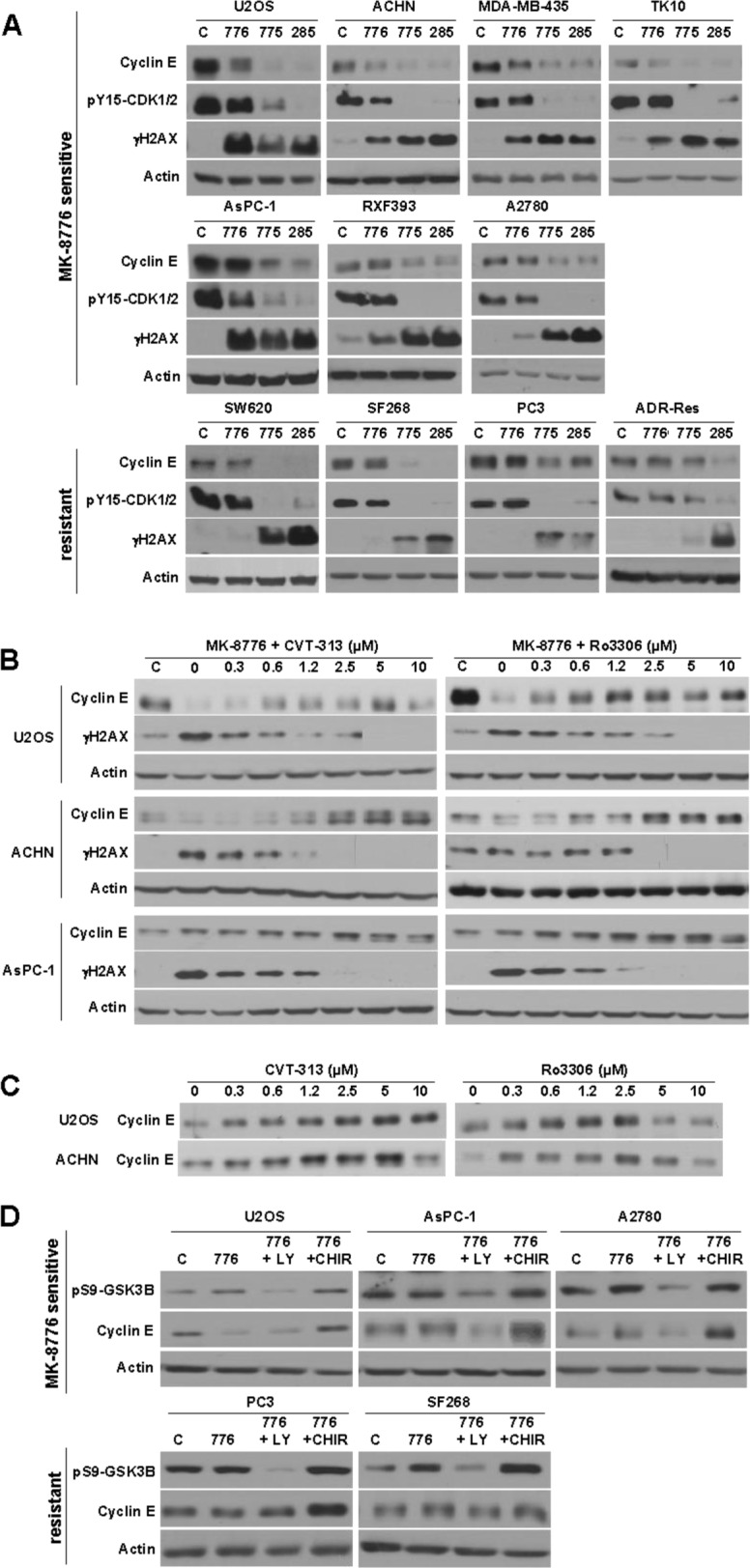
Modulation of cyclin E levels by MK-8776, AZD1775 and PD166285 **A.** The indicated cell lines were incubated with 2 μM of each drug for 6 h, then lysed and analyzed by western blotting. The top row reflects cells sensitive to MK-8776. The second row reflects cells that are also sensitive to MK-8776, but which fail to degrade cyclin E. The third row reflects MK-8776-resistant cell lines. **B.** The indicated cell lines were incubated concurrently with 2 μM MK-8776 and either 0 - 10 μM CVT-313 (left) or Ro3306 (right) for 6 h then analyzed by western blotting. **C.** Cells were incubated with 0 - 10 μM CVT-313 or Ro3306 alone for 6 h, then analyzed by western blotting. **D.** Cells were incubated with 2 μM MK-8776 for 6 h in the presence or absence of 10 μM LY294002 to inhibit PI3-kinase, or 5 μM CHIR-99021 to inhibit GSK3. Abbreviations: c, control (untreated); 776, MK-8776; 775, AZD1775; 285, PD166285; LY, LY294002; CHIR, CHIR-99021. Several images were reordered for clarity of presentation, but in each case the images were derived from the same exposure of the same western blot.

Activation of CDK1 or CDK2 is frequently assessed as dephosphorylation of tyrosine 15. Unfortunately, the phosphotyrosine-15-specific antibodies do not discriminate between CDK1 and CDK2 (despite what most suppliers state) as the phosphotyrosine resides in the middle of a 13 amino acid conserved sequence. We have confirmed this by selectively immunoprecipitating CDK2, and showing that a purported phosphotyrosine-15-CDK1 antibody detects the phosphorylated CDK2 (Supplemental Figure S3). The failure to detect dephosphorylation with MK-8776 (Figure 2; see also Figure 4A) might be explained by the continued presence of phospho-CDK1 masking any dephosphorylation of CDK2. It has been reported that CDK1 is present at 10-fold higher levels than CDK2 [21], so the phospho-CDK2 would represent a very small proportion of the total phospho-CDK1/2.

To better define the differential involvement of CDK1 *versus* CDK2, we used a small molecule inhibitor, CVT-313, which is reported to be about 10-fold more selective for CDK2 over CDK1 when tested against purified kinases [22]. We found that CVT-313 reduced the number of cells exhibiting γH2AX by 50% around 1 μM whereas it required about 10 μM to inhibit pHH3 by 50% (Figure 3). These results were similar whether γH2AX was induced by AZD1775 or MK-8776. Using the comet assay, we also demonstrated that CVT-313 prevented the appearance of MK-8776-induced DSB (Figure 2B).

To contrast these results, we also used Ro3306 which is reported to be about 10-fold more selective for CDK1 against the purified kinases [23]. However, Ro3306 inhibited both γH2AX and pHH3 at 2.5 μM suggesting that it does not discriminate between CDK1 and CDK2 in cells (Figure 3E, 3F). This inability of Ro3306 to preferentially inhibit CDK1 over CDK2 in cells may be attributable to the far lower level of active CDK2 compared to CDK1 in the cells as discussed above [21].

We further compared the efficacy of CVT-313 and Ro3306 in otherwise undamaged, but synchronized cells. CVT-313 was more effective at preventing progression through G1, but Ro3306 was about equipotent at inducing G1 and G2 arrest consistent with it inhibiting both CDK1 and CDK2 (Supplemental Figure S4). Importantly, neither inhibitor appeared to prevent progression through S phase.

The results with Ro3306 require additional comment as low concentrations caused an increase in pHH3 (Figure 3; Supplemental Figure S4) and an increase in the proportion of cells in G2/M, which we attribute to partial inhibition of CDK1 preventing complete passage through mitosis. The results with Ro3306 are clearly different than those obtained with CVT-313, and are consistent with the latter compound preferentially inhibiting CDK2. These data further support the model whereby γH2AX is a consequence of CDK2 activation, whereas pHH3 is a consequence of CDK1 activation. Importantly, MK-8776 did not activate CDK1 yet both CVT-313 and Ro3306 inhibited γH2AX at concentrations that implicate inhibition of CDK2.

### Cyclin E degradation as a marker of CDK2 activity

Neither HH3 nor H2AX is a direct phosphorylation target of CDK1 or CDK2. We therefore sought a more direct target. CDK2 complexes with cyclin E and, when activated, phosphorylates cyclin E resulting in its degradation [24, 25]. This is exactly what was observed in several sensitive cell lines (Figure 4A). For example, U2OS, ACHN, MDA-MB-435 and TK10 cells show degradation of cyclin E upon incubation with MK-8776 and AZD1775. The degradation of cyclin E was prevented by low concentrations of CVT-313 consistent with CDK2 inhibition (Figure 4B). Importantly, the results show the correlation between inhibition of γH2AX and the accumulation of cyclin E further supporting the premise that the DNA damage is a consequence of CDK2 activation.

Ro3306 also prevented the degradation of cyclin E and the appearance of γH2AX at ∼2.5 μM which is consistent with the data above suggesting that Ro3306 also inhibits CDK2 at this concentration. Interestingly, incubation of these cell lines with either CVT-313 or Ro3306 alone also induced accumulation of cyclin E (Figure 4C) suggesting that a basal level of CDK2 activity provides constitutive turnover of the protein.

Surprisingly, several of the sensitive cell lines (AsPC-1, RXF393 and A2780) did not decrease cyclin E upon incubation with MK-8776, although it was decreased by AZD1775 (Figure 4A). Degradation of cyclin E is not solely regulated by CDK2 but by a phosphodegron whereby CDK2 phosphorylates ser399 and GSK3B then phosphorylates thr395 [25]. Consequently, the degradation of cyclin E in U2OS cells was prevented by incubation with the GSK3B inhibitor CHIR-99021 (Figure 4D). We therefore questioned whether the failure to degrade cyclin E in AsPC1 and A2780 cells was due to a lack of active GSK3B. The cells were incubated with LY294002 to inhibit PI3K, which in turn leads to activation of GSK3B (confirmed by the decreased phosphorylation of ser9), and as a consequence, cyclin E was now degraded (Figure 4D). This degradation was also prevented by concurrent inhibition of GSK3B by CHIR-99021. These results suggest that the failure to degrade cyclin E is not a deficiency in activation of CDK2 but rather in the limited availability of active GSK3B.

**Figure 5:**
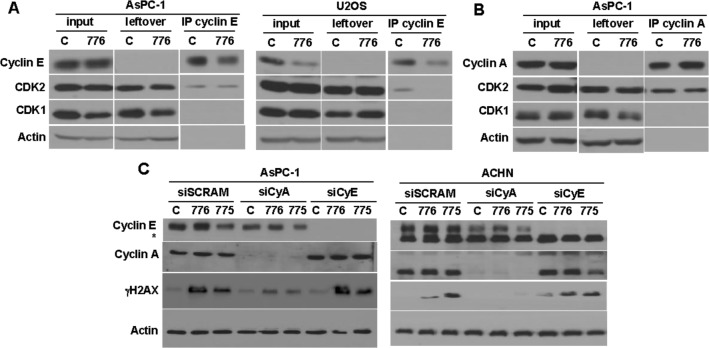
Role of cyclin E and cyclin A in inducing γH2AX **A.** AsPC-1 (left) and U2OS cells (right) were incubated with 2 μM MK-8776 for 6 h, or left untreated, then cyclin E was immunoprecipitated and the amount of associated CDK1 and CDK2 was assessed by western blotting. **B.** AsPC-1 cells were similarly treated but immunoprecipitation was performed with anti-cyclin A antibody. **C.** AsPC-1 (left) and ACHN (right) cells were transfected with siRNA targeting either cyclin A, cyclin E, or scrambled control. After 48 h, the cells were incubated with 2 μM MK-8776 or AZD1775 for 6 h, and assayed by western blotting for the amount of γH2AX. The asterisk identifies a non-specific band in the cyclin E blot for ACHN cells. Abbreviations: 776, MK-8776; 775, AZD1775; IP, immunoprecipitate.

To further dissect the regulation of cyclin E/CDK2 in the U2OS and AsPC-1 cells, we immunoprecipitated cyclin E and assessed the amount of CDK that co-immunoprecipitated. In both cell lines, a very low level of CDK2 was associated with cyclin E either constitutively or after incubation with MK-8776; no CDK1 was detected (Figure 5A). We also assessed the amount of CDK2 that co-immunoprecipated with cyclin A; more CDK2 was detected but most still appeared to be unbound (Figure 5B). These results suggest that the majority of CDK2 is not associated with either cyclin A or E and therefore inactive. These observations are attributed to the fact that, unlike CDK1/cyclin B, CDK2 is phosphorylated on tyrosine 15 independent of cyclin E or cyclin A, and only a small amount then complexes with the cyclins [26]. To confirm this hypothesis, we immunoprecipitated CDK2 to assess its phosphorylation status and indeed found that most of the CDK2 remained phosphorylated even when cells were incubated with MK-8776 (Supplemental Figure S3). In contrast, inhibition of Wee1 prevented the initial phosphorylation of CDK2 so no phosphorylation was detected. These observations also provide an additional explanation as to why little if any dephosphorylation of CDK2 was observed upon incubation of cells with MK-8776; as the majority of phospho-CDK2 is not bound to cyclin E or A, it is not subject to dephosphorylation by CDC25A.

### Resistance to MK-8776 is associated with failure to activate CDK2

The experiments above suggested that CDK2 activity is required for sensitivity of cells to both MK-8776 and AZD1775. We therefore selected several of the cell lines that were resistant to MK-8776 (Figure 1). These cells failed to dephosphorylate CDK1/2, did not degrade cyclin E, and did not accumulate γH2AX (Figure 4A). The failure to degrade cyclin E might be attributable to limited GSK3B activity as seen for AsPC1 cells. Incubation with LY294002 activated GSK3B as judged by decreased phosphorylation of ser9-GSK3, but unlike AsPC-1 cells, this failed to induce degradation of cyclin E, consistent with the failure of MK-8776 to activate CDK2 (Figure 4D).

**Figure 6:**
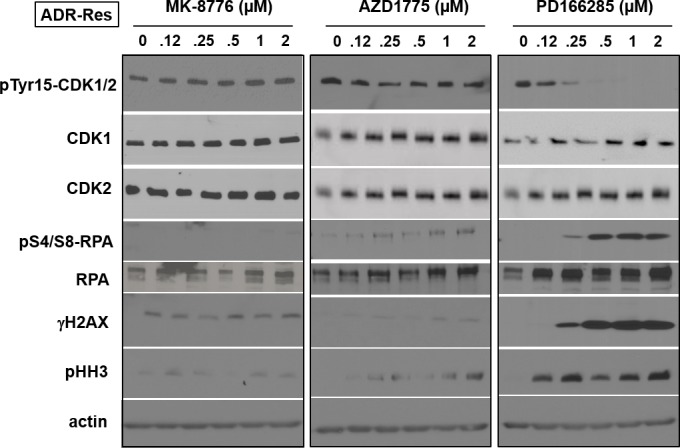
Mechanism of resistance of ADR-Res cells to MK-8776 ADR-Res cells were incubated with the indicated concentrations of either MK-8776, AZD1775 or PD166285 for 6 h, then analyzed by western blotting. These cells were only sensitive to PD166285 and only showed increased γH2AX and pHH3 at concentrations that inhibited pY15-CDK1/2.

ADR-Res cells were resistant to both MK-8776 and AZD1775, and this correlated with a failure to activate CDK2 as assessed by degradation of cyclin E (Figure 4A). AZD1775 also failed to induce dephosphorylation of CDK1/2 (Figures 4A and 6A). One possible mechanism of resistance to AZD1775 is that they rely on Myt1 to prevent activation of CDK1/2. We are unaware of any selective inhibitors of Myt1, but PD166285 is a broad tyrosine kinase inhibitor that inhibits both Wee1 and Myt1 [17]. All the cell lines were sensitive to a 24-h incubation with PD166285 (Figure 1C), and γH2AX was observed preferentially in S phase cells with potency similar to both MK-8776 and AZD1775 (Supplemental Figure S2C). ADR-Res cells incubated with PD166285 exhibited dephosphorylation of CDK1/2, and phosphorylation of RPA, H2AX and HH3 within 6 h (Figure 6). This acute sensitivity of ADR-Res cells suggests that resistance to AZD1775 might be mediated by Myt1.

### Cyclin A/CDK2 is responsible for the appearance of γH2AX

CDK2 normally partners with both cyclin E and cyclin A. The results above have correlated the activation of cyclin E/CDK2 with sensitivity to MK-8776 and AZD1775. However, the DSB could equally be the consequence of activation of cyclin A/CDK2, and CDK2 did immunoprecipitate with both cyclins (Figure 5A and 5B). To address this possibility, we transfected cells with siRNA targeting either cyclin E or cyclin A. In both AsPC-1 and ACHN cells, the suppression of cyclin A, but not cyclin E, resulted in suppression of γH2AX (Figure 5C). As CDK1 did not immunoprecipitate with cyclin A (Figure 5B), these results support the contention that cyclin A/CDK2 activation is critical for the onset of DSB.

### MK-8776-mediated accumulation of CDC25A predicts sensitivity to MK-8776

**Figure 7:**
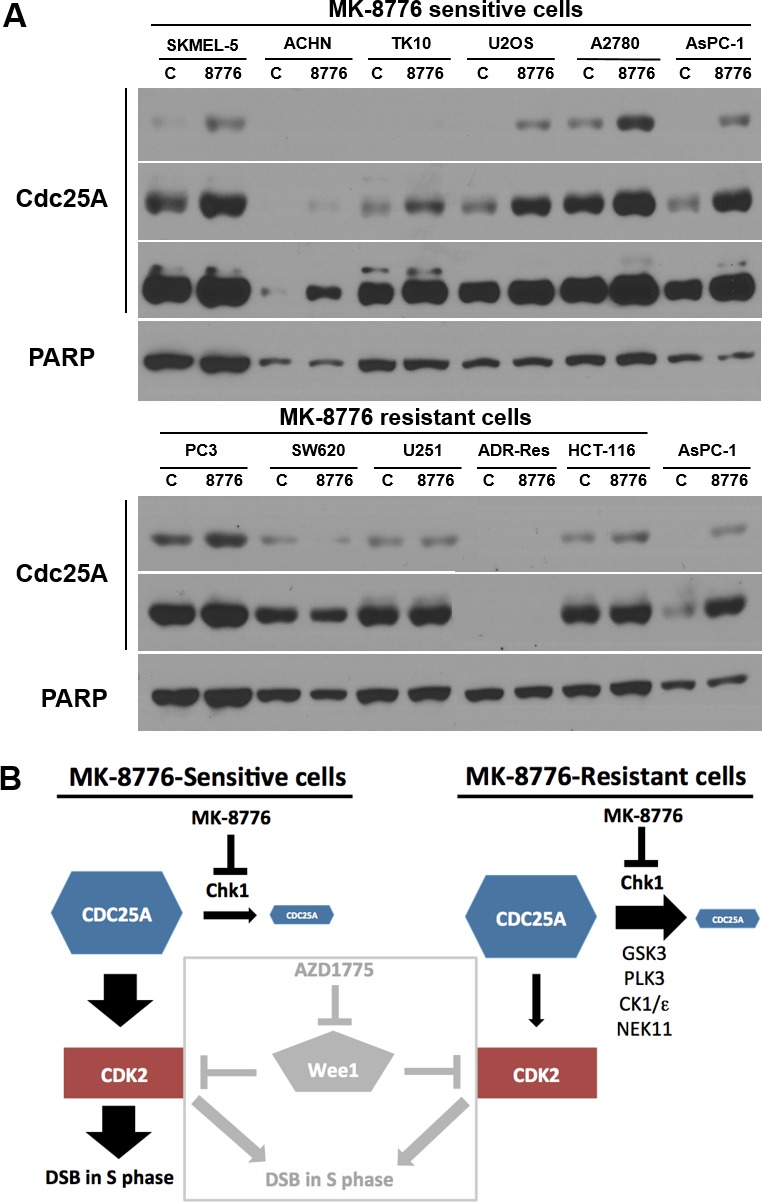
Impact of MK-8776 on CDC25A levels in sensitive and resistant cells **A.** Sensitive (top) and resistant cells (bottom) were incubated with MK-8776 for 6 h, then analyzed by western blotting for changes in level of CDC25A protein. AsPC-1 cells are included in both panels for comparison. Several exposures are shown of each blot. Each lane contained lysate from 20,000 cells. PARP was used as a loading control; while it differs in level between cell lines, it demonstrates equal loading between control and treated samples for each line. **B.** Model of the signaling pathway impacted by Chk1 in MK-8776-sensitive and resistant cells. A role for any of the alternate kinases (GSK3, PLK3, CK1/ε and NEK11) in eliciting resistance remains to be established. The box demonstrates how the Wee1 inhibitor AZD1775 activates CDK2 in both MK-8776-sensitive and resistant cells.

The activation of CDK2 is a consequence of its dephosphorylation by CDC25A which, in turn, is a direct target of Chk1. Phosphorylation by Chk1 causes degradation of CDC25A and maintains low basal levels of CDC25A [27]. Therefore, we assessed the impact of MK-8776 on the levels of CDC25A. In six sensitive cell lines, MK-8776 induced accumulation of CDC25A, consistent with the observed activation of CDK2 (Figure 7A). In contrast, five resistant cell lines exhibited no change in CDC25A levels consistent with the failure of MK-8776 to activate CDK2. There was a large range of levels of CDC25A across the cell lines requiring multiple exposures of the western blots to show the differences; one cell line, ADR-Res, expressed no detectable protein (note the AsPC-1 lysate is shown in both blots for direct comparison of levels). Interestingly, there is no correlation between basal CDC25A levels and sensitivity to MK-8776.

## DISCUSSION

By screening a large number of cell lines, we have established that only about 15% are hypersensitive to the Chk1 inhibitor MK-8776 following a 24-h incubation (IC50 < 2 μM). Our cytotoxicity assay differs from more commonly used continuous incubations of cells with drug as it assesses the ability of cells to recover over 6 days following a short incubation. In this regard, it more closely approximates a clonogenic assay. Furthermore, the short incubation with drug might better reflect what can be achieved upon administration to a patient. It also demonstrates a potential therapeutic window that is based on both dose and time of administration. For example, a non-transformed, but immortalized breast cell line (IMEC) was completely resistant to a 48-h incubation and only showed a slight growth inhibition after 7 days. MK-8776 continued to inhibit Chk1 in resistant cells for 7 days without impeding their growth. This differs from experiments in mice in which deletion of Chk1 led to embryonic lethality [28, 29]. Previous experiments have been performed with U2OS cells that are sensitive to Chk1 inhibition [30], but those studies failed to recognize that the majority of cell lines can tolerate inhibition of Chk1 for long periods.

Sensitivity to Chk1 inhibition was observed as rapid induction (< 6 h) of γH2AX in S phase cells. As more cells progress from G1 into S, they also succumb to Chk1 inhibition and exhibit high γH2AX. Prior experiments have demonstrated that the γH2AX in U2OS cells correlates with the appearance of DSB [11], and this was confirmed for AsPC-1 cells in this study. These DSB were prevented by CVT-313 at concentrations that selectively inhibit CDK2, and by siRNA-mediated suppression of cyclin A. CDK2 is required for replication origin firing and is therefore suppressed by Chk1 [31]. Chk1 inhibition in early S phase leads to premature firing of late replication origins, which may result in collision of these late replicons with ongoing transcription, R loop formation and replication fork collapse [32]. Whether this pathway is involved in the observed cytotoxicity remains to be determined, but downstream effectors, Mre11 and Mus81 nucleases, have been implicated in the eventual DNA breakage [11, 33, 34].

It was unexpected to find that cells do not tolerate increased CDK2 activity in S phase, although, in retrospect, this has been reported previously using a CDK2 variant that can not be phosphorylated on thr14 and tyr15 [35]. In contrast, CDK2 is usually considered essential for progression through S phase, as it is required for loading Cdc45 on to DNA to initiate the firing of each replication fork [31]. However, we observed that incubation of undamaged cells with CVT-313 failed to arrest cells in S phase suggesting that this requirement for CDK2 can be circumvented. Incubation of undamaged cells with CVT-313 did cause accumulation of cyclin E suggesting that there is sufficient constitutive CDK2 activity to limit cyclin E/CDK2. It is possible that there is a threshold level of activity of CDK2 below which cells survive, but which, once exceeded, leads to DSB. Cells may activate CDK2 in S phase as a stress response that permits circumvention of blocked replication by activating alternate replicons. Hence, there must be a careful balance between the CDK2 activity required for replication recovery *versus* induction of extensive DNA fragmentation.

The majority of cell lines were resistant to Chk1 inhibition. Resistance did not correlate with any particular organ site or p53 status. While the observed DSB might induce a p53 response, that would occur after activation of CDK2 so could not prevent the damage. Several cell lines had been selected for other phenotypes that might contribute to sensitivity (e.g., 2008, a FANCF-defective ovarian line; ATLD1, isolated from a patient defective for Mre11), but they, and their complemented derivatives, remained resistant to MK-8776 in our analysis (Figure 1).

To begin to understand the mechanism of resistance to MK-8776, we asked whether more direct activation of CDK2 using the Wee1 inhibitor AZD1775 would circumvent resistance. Inhibition of Wee1 was cytotoxic to most of the cell lines, and the few resistant lines were sensitive to dual inhibition of Wee1 and Myt1. MK-8776-resistant cells failed to activate CDK2 or degrade cyclin E (a consequence of CDK2 activation) in response to Chk1 inhibition. This compared to the sensitive cells that activated CDK2 and, in most cases, degraded cyclin E. Several sensitive cells failed to degrade cyclin E, but this was attributed to limited activity of GSK3B, whose activity is also required to phosphorylate the phosphodegron in cyclin E that leads to its degradation. In contrast, activation of GSK3B in resistant cells, in concert with MK-8776, did not lead to degradation of cyclin E. These results suggest that the mechanism of resistance to MK-8776 is mediated upstream of CDK2 activation (Figure 7B). In support of this conclusion, MK-8776 induced accumulation of CDC25A only in the sensitive cells. CDC25A regulation is complex with input from multiple other kinases resulting in either degradation or sequestration depending on cell cycle phase [36-39]. Future studies will be directed to defining the critical proteins that regulate CDC25A and discriminate sensitive and resistant cells.

It has previously been demonstrated that the Wee1 inhibitor can force S phase-arrested cells to directly enter mitosis without completing DNA synthesis, albeit only in some cell lines [5, 14]. It was suggested this was a novel mechanism of action whereby unscheduled entry into mitosis explained the underlying sensitivity to AZD1775. However, we demonstrate that extensive γH2AX can occur in S phase upon incubation with either MK-8776 or AZD1775, and these cells are distinct from those positive for pHH3, demonstrating that mitotic events are not required for the DSB observed here.

These experiments required a critical reevaluation of the tools available to discriminate CDK1 from CDK2. Many suppliers provide antibodies that are purported to detect phosphotyrosine on CDK1, while other antibodies are purported to detect the phosphotyrosine on CDK2. However, this is a highly conserved epitope in both proteins and the antibodies do not discriminate the two kinases. The use of siRNA to suppress one or other kinase can also be misleading as this can arrest cell cycle progression in G1 or G2 and thereby protect cells from a drug that requires S phase progression. We found that the commonly used CDK1-selective inhibitor Ro3306 was equally effective at inhibiting CDK2. This observation may relate to the much lower level of CDK2 present in cells compared to CDK1, and also to the very small proportion of CDK2 that complexes with either cyclin E or cyclin A [21, 26]. We conclude that CVT-313 is more selective for CDK2 and this was a major tool to help understand the critical role of CDK2. Many substrates can be phosphorylated by both CDK1 and CDK2, but cyclin E should be a selective target for CDK2 as they form a heterodimeric complex. The fact that cyclin E was degraded whenever CDK2 was active was also a useful tool in these studies, but with the proviso that concurrent GSK3B-mediated phosphorylation is also required for cyclin E degradation. An interesting extrapolation of these observations is that cells or tumors with elevated cyclin E protein probably reflect cells with inactive CDK2.

Several Chk1 inhibitors have been combined with DNA damaging agents in clinical trials, though several were terminated because of toxicity [2, 40]. Many of the drugs are not pure Chk1 inhibitors, so it is not clear whether the toxicity is due to off-target effects. For example, a recent report showed that three Chk1 inhibitors also inhibit FLT3 [41]. MK-8776 is possibly one of the most selective inhibitors, and clinical trials have been performed in combination with both cytarabine and gemcitabine; partial responses were observed but further development has been terminated for business reasons [15, 42, 43]. A Phase I clinical trial of GDC0425 plus gemcitabine continues (www.clinicaltrials.gov) though the selectivity of this compound has not yet been reported. The only clinical trial testing a Chk1 inhibitor as monotherapy involves LY2606368, although this compound also inhibits a variety of other kinases including Chk2 and RSK1-3. Unfortunately no attempt has been made to stratify patients who might respond. This is critical if Chk1 inhibitors are to succeed as monotherapy.

In summary, we conclude that CDK2 is usually repressed in S phase cells, and its untimely activation explains the sensitivity to Chk1 and Wee1 inhibitors as single agents. The mechanism of resistance to MK-8776 appears to rely on redundant pathways that regulate CDC25A, whereas the sensitive cells appear to rely solely on Chk1. Identification of the critical determinants of response is required so that appropriate patients can be stratified to clinical trials of Chk1 inhibitors as monotherapy.

## MATERIALS AND METHODS

### Cell culture

The majority of cell lines were derived from the NCI-60 panel and obtained from the Developmental Therapeutics Program, National Cancer Institute. Other cell lines were obtained from the American Type Culture Collection, or described in our previous papers [3, 11]. Cells were maintained in RPMI1640 plus 10% fetal bovine serum, antibiotic and antimycotic.

### Chemicals

MK-8776 and AZD1775 were provided by Merck. PD166285, CVT-313, Ro3306, LY294002 and CHIR-99021 were obtained from Sigma. SN38 was provided by Pfizer. Stock solutions were made at 10 mM in dimethylsulfoxide (CHIR-99021 at 5 mM; SN38 100 μM). SMART pool siRNA for cyclin E (CCNE1) and cyclin A2 (CCNA2) were obtained from Dharmacon.

### Growth inhibition assays

Inhibition of cell growth was assessed by plating 500 - 5000 cells (depending on growth rate) per well of a 96-well plate. The following day, drugs were added as 2-fold dilutions from 10 μM (8 wells/concentration). After 24 and 48 h, drug was removed, and replaced with fresh media. A third plate was continuously incubated with drug. Before reaching confluence (6 - 7 days), cells were washed, lysed and stained with Hoechst 33258 as previously described [3, 44]. Fluorescence was read on a microplate spectrofluorometer.

### Antibodies and immunoblotting

For protein analysis, cells were incubated with drugs in 6-well plates, rinsed, lysed in Laemmli sample buffer and boiled for 5 min. Proteins were separated by SDS-PAGE and transferred to polyvinylidene difluoride membranes. Western blotting was performed with the following antibodies: p-Y15-CDK1 (also detects phospho-CDK2 (cst-9111)), p296-Chk1 (cst-2349), γH2AX (cst-9718), cyclin A2 (cst-4656), pS9-GSK3B (cst-9331), pHH3 (cst-3377) (Cell Signaling Technology); Chk1 (sc-8404), cyclin E (sc-247) (Santa Cruz Biotechnology Inc.); CDK1 (8878), CDK2 (05-596) (Millipore); CDC25A (ms-638; Thermo Scientific); RPA (ab-2175; Abcam); pS4/S8-RPA (NBP 1-23017; Novus); actin (A3854; Sigma).

### Immunoprecipitation

Cells were lysed in lysis/wash buffer provided in the Classic Magnetic IP/Co-IP Kit (Pierce, 8804) with added protease and phosphatase inhibitors for 30 min on ice, then centrifuged at 13,000g for 10 min. The extract (500 μg) was pre-cleared with Protein A/G magnetic beads for 1 h at 4°C and the supernatant was collected. Antibody [2.5 μg; cyclin E (sc-198), cyclin A (sc-751)] was added to the supernatant and mixed for 3 h at 4°C. Pre-washed magnetic beads were added and incubated on a rotator at 4°C overnight. The supernatant was recovered, and the immunoprecipitate was washed and resuspended in 2 x Laemmli sample buffer. Equivalent portions of supernatant and immunoprecipitate were subjected to 10% polyacrylamide SDS-PAGE and immunoblotting performed for the proteins indicated.

### Cell cycle analysis

Cell cycle analysis was conducted by flow cytometry using propidium iodide as described previously [4]. For 2-dimensional flow cytometry, cells were also labeled with Alexa 488-conjugated γH2AX or pHH3 (Cell Signaling Technology). Cells were analyzed on either a Becton Dickinson FACScan or FACScalibur flow cytometer.

### Comet assay

AsPC-1 cells were incubated with MK-8776 alone, or in combination with CVT-313 for 6 hours.

Preparation and execution of the neutral comet assay was done according to the Trevigen Comet Assay protocol for single-cell gel electrophoresis. The slides were imaged with a fluorescent microscope, and comet tails were scored using ImageJ software. The tail moment for the control was calculated and a threshold value set at 1 standard deviation above the mean. Results are expressed as the percent of cells with a tail moment above 1SD of the control.

## SUPPLEMENTARY MATERIAL FIGURES



## References

[1] Boutros R, Lobjois V, Ducommun B (2007). CDC25 phosphatases in cancer cells: key players? Good targets?. Nature Rev Cancer.

[2] Thompson R, Eastman A (2013). The cancer chemotherapeutic potential of Chk1 inhibitors: how mechanistic studies impact clinical trial design. Br J Clin Pharmacol.

[3] Montano R, Chung I, Garner KM, Parry D, Eastman A (2012). Preclinical development of the novel Chk1 inhibitor SCH900776 in combination with DNA damaging agents and antimetabolites. Mol Cancer Therap.

[4] Montano R, Thompson R, Chung I, Hou H, Khan N, Eastman A (2013). Sensitization of human cancer cells to gemcitabine by the Chk1 inhibitor MK-8776: cell cycle perturbation and impact of administration schedule *in vitro* and *in vivo*. BMC Cancer.

[5] Aarts M, Sharpe R, Garcia-Murillas I, Gevensleben H, Hurd MS, Shumway SD, Toniatti C, Ashworth A, Turner NC (2012). Forced mitotic entry of S phase cells as a therapeuutic strategy induced by inhibition of WEE1. Cancer Discovery.

[6] Brana I, Mackay H (2014). WEE1 inhibition as anticancer strategy: first advances. Drugs of the Future.

[7] Guzi T, Paruch K, Dwyer MP, Labroli M, Shanahan F, Davis N, Taricani L, Wiswell D, Seghezzi W, Penaflor E, Bhagwat B, Wang W, Gu D, Hsieh Y, Lee S, Liu M, Parry D (2011). Targeting the replication checkpoint using SCH 900776, a potent and selective CHK1 inhibitor identified *via* high content functional screening. Mol Cancer Therap.

[8] Davies KD, Cable PL, Garrus JE, Sullivan FX, von Carlowitz I, Huerou YL, Wallace E, Woessner RD, Gross S (2011). Chk1 inhibition and Wee1 inhibition combine synergistically to impede cellular proliferation. Cancer Biol Therap.

[9] Carrassa L, Chila R, Lupi M, Ricci F, Celenza C, Mazzoletti M, Broggini M, Damia G (2012). Combined inhibition of Chk1 and Wee1. *In vitro* synergistic effect translates to tumor growth inhibition *in vivo*. Cell Cycle.

[10] Guertin AD, Martin MM, Roberts B, Hurd M, Qu X, Miselis NR, Liu Y, Li J, Benita Y, Bloecher A, Toniatti C, Shumway SD (2013). Unique functions of CHK1 and WEE1 underlie synergistic anti-tumor activity upon pharmacologic inhibition. Cancer Cell Internatl.

[11] Thompson R, Montano R, Eastman A (2012). The Mre11 nuclease is critical for sensitivity of cells to Chk1 inhibition. PLoS One.

[12] Zeman MK, Cimprich KA (2014). Causes and consequences of replication stress. Nature Cell Biol.

[13] Murga M, Campaner S, Lopez-Contreras AJ, Toldeo LI, Soria R, Montano MF, D'Artista LD, Schleker T, Guerra C, Garcia E, Barbacid M, Hidalgo M, Amati B, Fernandez-Capetillo O (2011). Exploiting oncogene-induced replicative stress for the selective killing of Myc-driven tumors. Nature Structural and Molecular Biology.

[14] Aarts M, Bajrami I, Herrera-Abreu MT, Elliott R, Brough R, Ashworth A, Lord CJ, Turner NC (2015). Functional genetic screen identifies increased sensitivity to WEE1 inhibition in cells with defects in Fanconi anemia and HR pathways. Mol Cancer Therap.

[15] Daud AI, Ashworth MT, Strosberg J, Goldman JW, Mendelson D, Springett G, Venook AP, Loechner S, Rosen L, Shanahan F, Parry D, Shumway S, Grabowsky JA, Freshwater T, Sorge C, Kang SP, Isaacs R, Munster PN (2015). A Phase I dose-escalation trial of Checkpoint kinase 1 inhibitor MK-8776 as monotherapy and in combination with gemcitabine in patients with advanced solid tumors. J Clin Oncol.

[16] Kohn EA, Ruth ND, Brown MK, Livingstone M, Eastman A (2002). Abrogation of the S phase DNA damage checkpoint results in S phase progression or premature mitosis depending on the concentration of UCN-01 and the kinetics of Cdc25C activation. J Biol Chem.

[17] Wang Y, Li J, Booher RN, Kraker A, Lawrence T, Leopold WR, Sun Y (2001). Radiosensitization of p53 mutant cells by PD0166285, a novel G2 checkpoint abrogator. Cancer Res.

[18] Zellweger R, Dalcher D, Mutreja K, Berti M, Schmid JA, Herrador R, Vindigni A, Lopes M (2015). Rad51-mediated replication fork reversal is a global response to genotoxic treatments in human cells. J Cell Biol.

[19] Zuazua-Villar P, Rodriguez R, Gagou ME, Eyers PA, Meuth M (2014). DNA replication stress in Chk1-depleted tumor cells trriggers premature (S-phase) mitosis through inappropriate activation of Aurora kinase B. Cell Death Disease.

[20] Syljuasen RG, Sorensen CS, Hansen LT, Fugger K, Lundiin C, Johansson F, Helleday T, Sehested M, Lukas J, Bartek J (2005). Inhibition of human Chk1 causes increased initiation of DNA replication, phosphporylation of ATR targets, and DNA breakage. Mol Cell Biol.

[21] Arooz T, Yam CH, Siu WY, Lau A, Li KKW, Poon RYC (2000). On the concentrations of cyclins and cyclin-dependent kinases in extracts of cultured human cells. Biochemistry.

[22] Brooks EE, Gray NS, Joly A, Kewar SS, Lum R, Mackman RL, Norman TC, Rosete J, Rowe M, Schow SR, Schultz PG, Wang X, Wick MM, Shiffman D (1997). CVT-313, a specific and potent inhibitor of CDK2 that prevents neointimal proliferation. J Biol Chem.

[23] Vassilev LT, Tovar C, Chen S, Knezevic D, Zhao X, Sun H, Heimbrook DC, Chen L (2006). Selective small-molecule inhibitor reveals critical mitotic functions of CDK1. Proc Natl Acad Sci USA.

[24] Clurman BE, Sheaff RJ, Thress K, Groudine M, Roberts JM (1996). Turnover of cyclin E by the ubiquitin-proteasome pathway is regulated by Cdk2 binding and cyclin phosphorylation. Genes Dev.

[25] Welcker M, Singer J, Loeb KR, Grim J, Bloecher A, Gurien-West M, Clurman BE, Roberts JM (2003). Multisite phosphorylation by CDK2 and GSK3 controls controls cyclin E degradation. Mol Cell.

[26] Coulonval K, Bockstaele L, Paternot S, Roger PP (2003). Phosphorylations of cyclin-dependent kinase 2 revisited using two-dimensional gel electrophoresis. J Biol Chem.

[27] Sorensen CS, Syljuasen RJ, Falck J, Schroeder T, Ronnstrand L, Khanna KK, Zhou B-B, Bartek J, Lukas J (2003). Chk1 regulates the S phase checkpoint by coupling the physiological turnover and ionizing radiation-induced accelerated proteolysis of Cdc25A. Cancer Cell.

[28] Liu Q, Guntuku S, Cui X-S, Matsuoka S, Cortez D, Tamai K, Luo G, Carattini-Rivera S, DeMayo F, Bradley A, Donehower LA, Elledge SJ (2000). Chk1 is an essential kinase that is regulated by ATR and required for the G2/M DNA damage checkpoint. Genes Dev.

[29] Takai H, Tominaga K, Motoyama N, Minamishima YA, Nagahama H, Tsukiyama T, Ikeda K, Nakayama K, Nakanishi M (2000). Aberrant cell cycle checkpoint function and early embryonic death in Chk1−/− mice. Genes Dev.

[30] Beck H, Nahse V, Larsen MSY, Groth P, Clancy T, Lees M, Jorgensen M, Helleday T, Syljuasen RG, Sorenson CS (2010). Regulators of cyclin-dependent kinases are crucial for maintaining genome integrity in S phase. J Cell Biol.

[31] Jones RM, Petermann E, Mizuno T, Hanaoka F, Nakanishi M (2012). Replication fork dynamics and the DNA damage response. Biochem J.

[32] Aguilera A, Garcia-Muse T (2012). R loops: from transcription byproducts to threats to genome stability. Mol Cell.

[33] Forment JV, Blasius M, Guerini I, Jackson SP (2011). Structure-specific DNA endonuclease Mus81/Eme1 generates DNA damage caused by Chk1 inactivation. PLoS One.

[34] Dominguez-Kelly R, Martin Y, Koundrioukoff S, Tanenbaum ME, Smits VAJ, Medema RH, Debatisse M, Freire R (2011). Wee1 controls genomic stability during replication by regulating the Mus81-Eme1 endonuclease. J Cell Biol.

[35] Hughes BT, Sidorova J, Swanger J, Monnat RJ, Clurman BE (2013). Essential role for CDK2 inhibitory phosphorylation during replication stress by a Cdk2 knockin mutation. Proc Natl Acad Sci USA.

[36] Kang T, Wei Y, Honaker Y, Yamaguchi H, Appella E, Hung MC, Piwnica-Worms H (2008). GSK-3B targets Cdc25A for ubiquitin-mediated proteolysis, and GSK-3B inactivation correlates with Cdc25A overproduction in human cancers. Cancer Cell.

[37] Sorensen CS, Melixetian M, Klein DK, Helin K (2010). NEK11. Linking Chk1 and CDC25A in DNA damage checkpoint signaling. Cell Cycle.

[38] Honaker Y, Piwnica-Worms H (2010). Casein kinse 1 functions as both penultimate and ultimate kinase in regulating Cdc25A destruction. Oncogene.

[39] Chen MS, Ryan CE, Piwnica-Worms H (2003). Chk1 kinase negatively regulates mitotic function of Cdc25A through 14-3-3 binding. Mol Cell Biol.

[40] Chen T, Stephens PA, Middleton FK, Curtin NJ (2012). Targeting the S and G2 checkpoint to treat cancer. Drug Discovery Today.

[41] Yuan LL, Green A, David L, Dozier C, Recher C, Didier C, Tamburini J, Manenti S (2014). Targeting CHK1 inhibits cell proliferation in FLT3-ITD positive acute myeloid leukemia. Leukemia Res.

[42] Karp JE, Thomas BM, Greer JM, Sorge C, Gore SD, Pratz KW, Smith BD, Flatten KS, Peterson K, Schneider P, Mackey K, Freshwater T, Levis MJ, McDevitt MA, Carraway HE, Gladstone DE, Showel MM, Loechner S, Parry DA, Horowitz JA, Isaacs R, Kaufmannn SH (2012). Phase I and pharmacologic trial of cytosine arabinoside with the selective checkpoint I inhibitor SCH 900776 in refractory acute leukemias. Clin Cancer Res.

[43] Sakurikar N, Eastman A (2015). Will targeting Chk1 have a role in the future of cancer therapy?. J Clin Oncol.

[44] Rao J, Otto WR (1992). Fluorometric DNA assay for cell growth estimation. Anal Biochem.

