# Supplementary material for: A subset of cancer cell lines is acutely sensitive to the Chk1 inhibitor MK-8776 as monotherapy due to CDK2 activation in S phase
Source: Oncotarget. 2015 Nov 22;7(2):1380–94. doi: 10.18632/oncotarget.6364 (PMC4811467; doi:10.18632/oncotarget.6364)
Supplement: Supplementary file 1 [file oncotarget-07-1380-s001.pdf]

A subset of cancer cell lines is acutely sensitive to the Chk1 inhibitor MK-8776 as monotherapy due to CDK2 activation in S phase

Supplementary Material

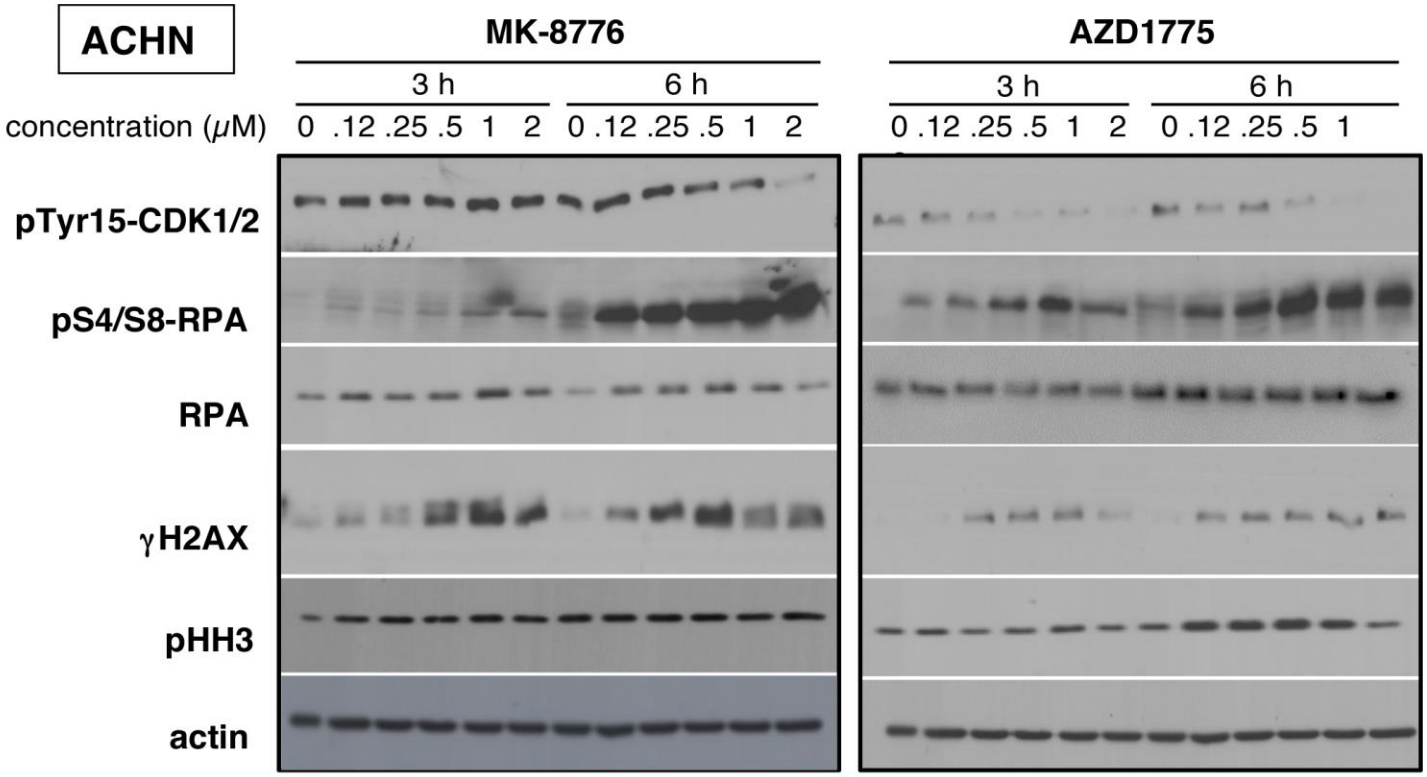

Supplemental Figure S1. Impact of MK-8776 and AZD1775 on markers of CDK1/2 activity and DNA double-strand breaks. ACHN cells were incubated with the indicated concentrations of MK-8776 and AZD1775 for 3 or 6 h, then lysed and analyzed by western blotting for the indicated proteins. Results are comparable to those shown for AsPC-1 cells in Figure 2.

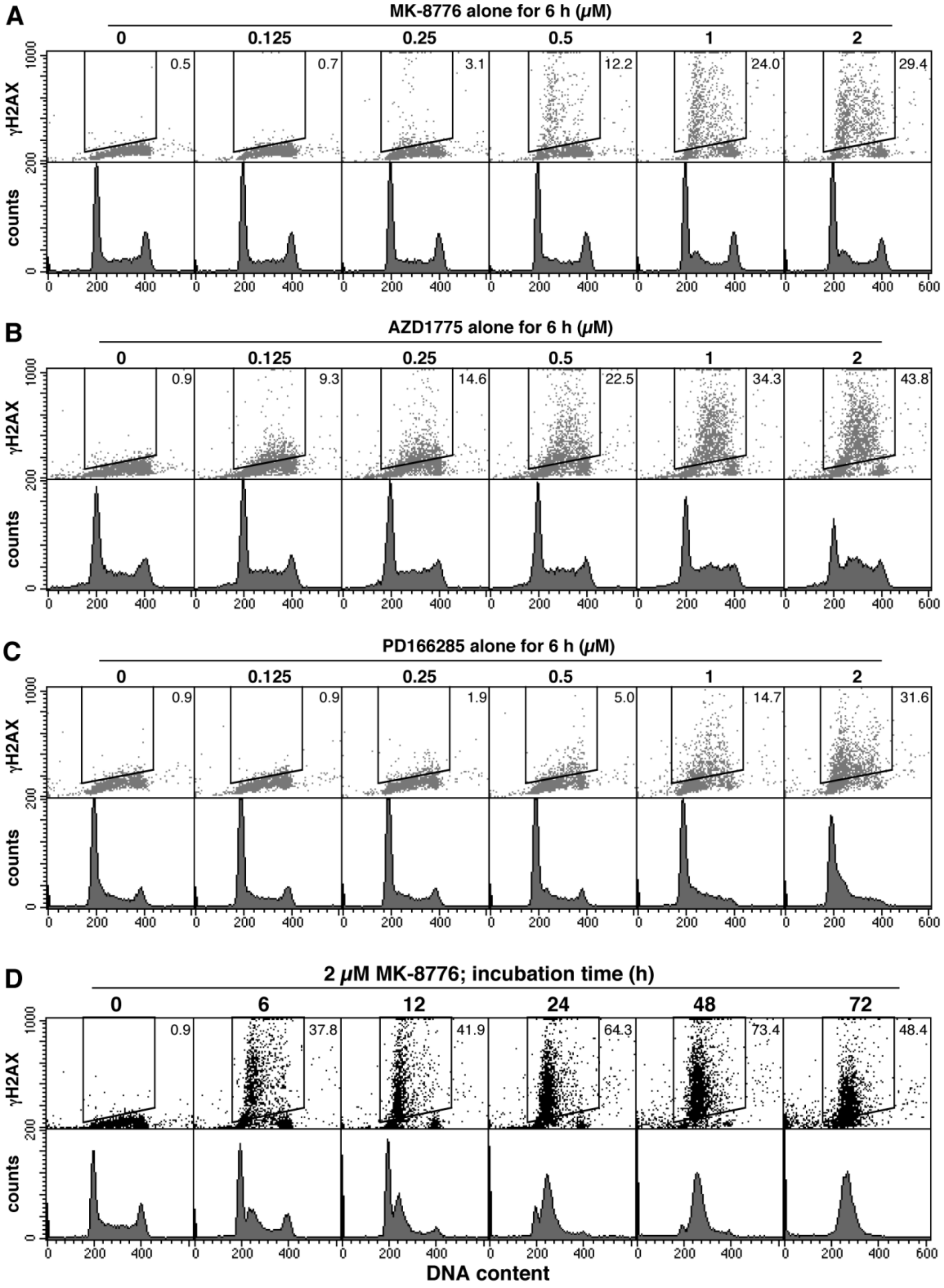

Supplementary Figure S1

Supplemental Figure S2. Impact of concentration and time on γH2AX induced by MK-8776, AZD1775 and PD166285. A-C, AsPC-1 cells were incubated with the indicated concentration of each drug for 6 h and analyzed by 2-dimensional flow cytometry for the extent of γH2AX at each phase of the cell cycle. D, AsPC-1 cells were incubated with 2 μM MK-8776 for 0 – 96 h then analyzed by flow cytometry.

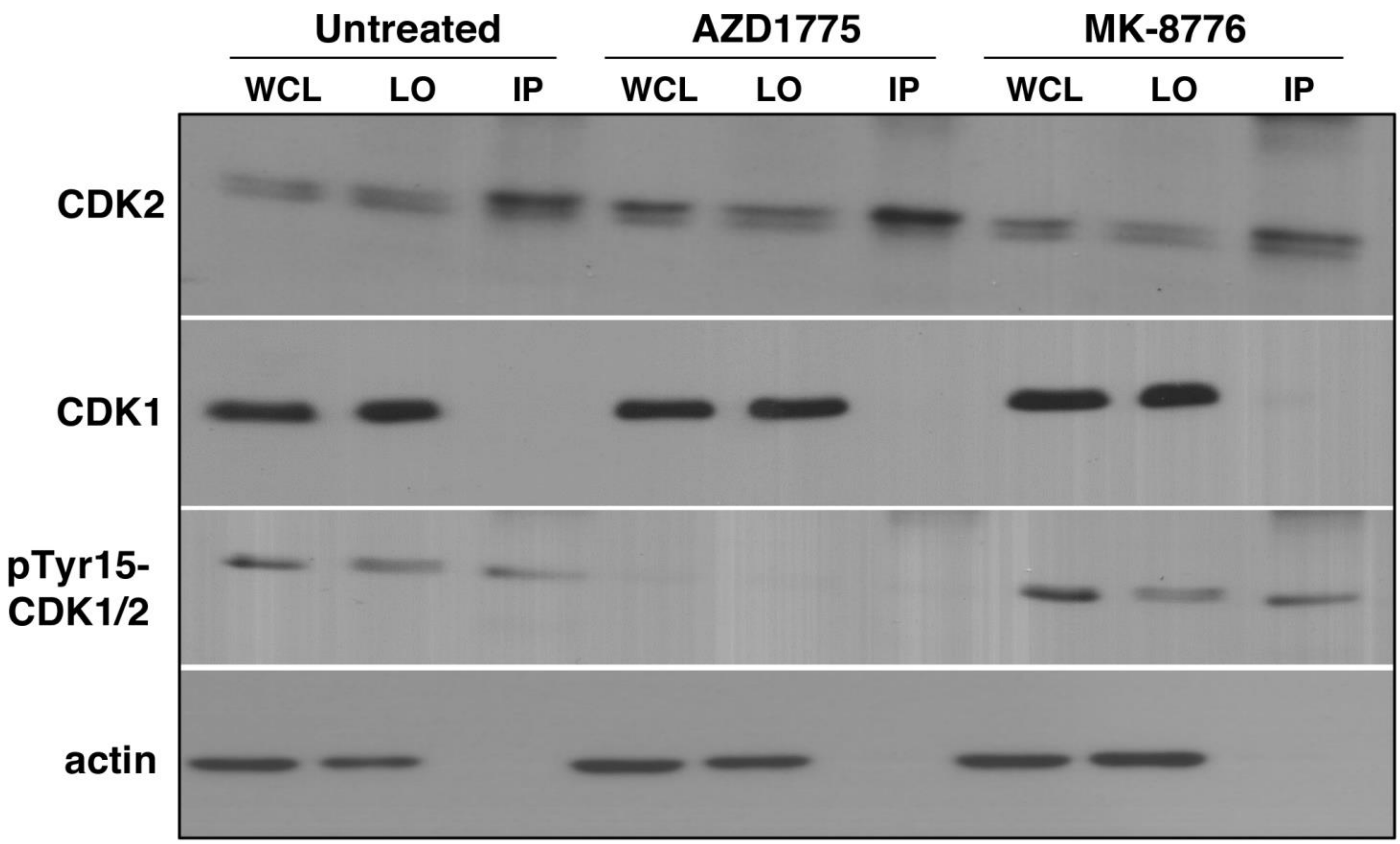

Supplemental Figure S3. Characterization of the antibody that detects phosphorylation at Y-15 on CDK1 and CDK2. U2OS cells were incubated with 1 μM AZD1775 or MK-8776 for 6 h, then lysed and immunoprecipitated for CDK2. No CDK1 was co-immunoprecipitated, yet the pellet was clearly positive for phosphorylated CDK (i.e., CDK2). Incubation with AZD1775 prevented phosphorylation at Y-15. Incubation with MK-8776 did not result in loss of pY-CDK2 because much of it is not complexed to cyclin E or A as discussed in the text.

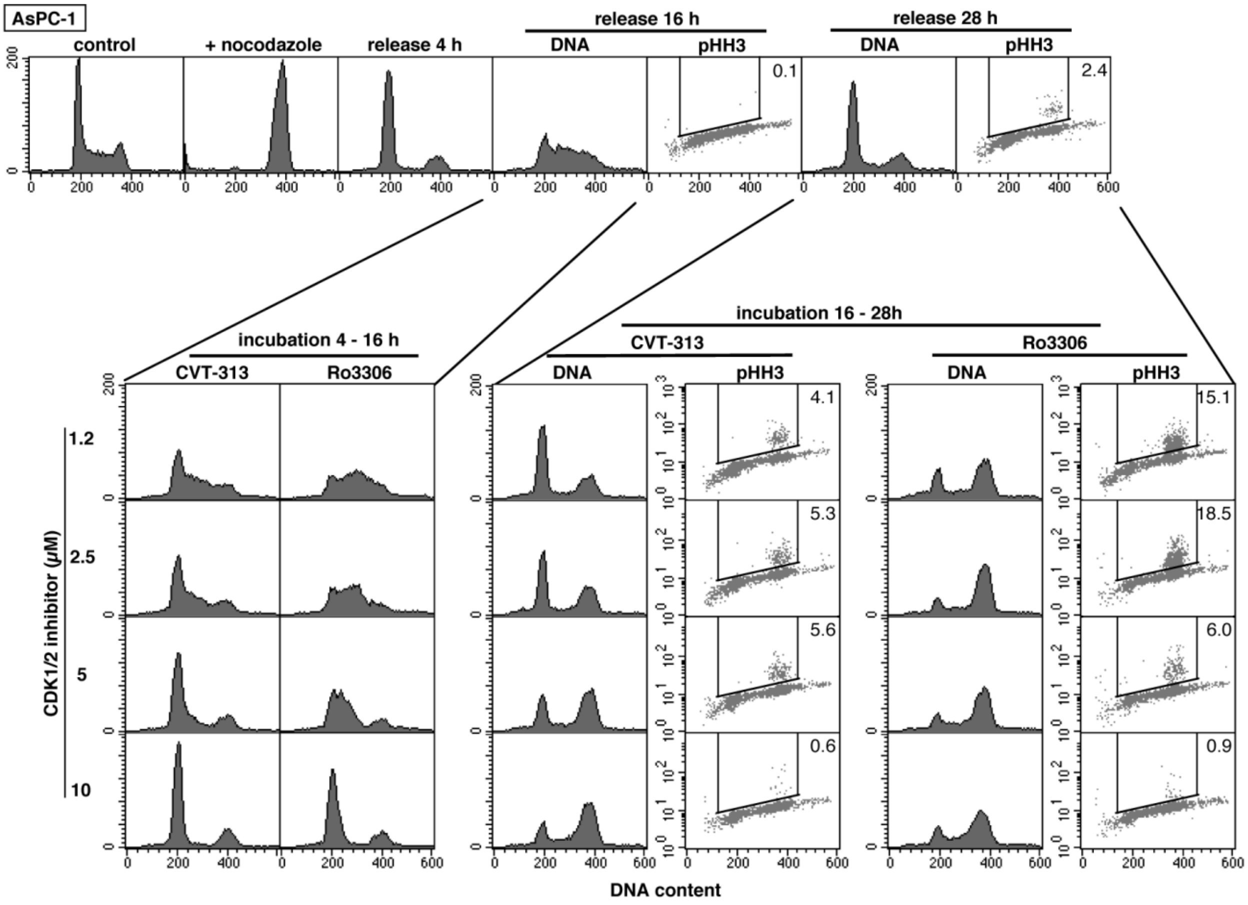

Supplemental Figure S4. Impact of CDK inhibitors on progression of undamaged cells through the cell cycle. AsPc-1 cells were synchronized overnight by incubation with 0.5 µg/ml nocodazole. Mitotic cells were harvested by shaking and plated in 6-well plates for 4 h, at which time, most of the cells had entered G1. The media was replaced and cells incubated for an additional 12 h (16 h time point; cells predominantly in S phase) or for 24 h (28 h time point; cells predominantly in the next G1). CVT313 and Ro3306 were added from either 4-16 h (left) or 16-28 h (right), harvested and analyzed by flow cytometry. In addition, cells harvested at 28 h were analyzed for pHH3 as an assessment of their arrest in G_2 (pHH3 negative) versus progression into M (pHH3 positive). The inset numbers reflect the percent of cells positive for pHH3.
